# The Impact of Ethnic-Immigrant Status and Obesity-Related Risk Factors on Behavioral Problems among US Children and Adolescents

**DOI:** 10.6064/2012/648152

**Published:** 2012-12-31

**Authors:** Gopal K. Singh, Stella M. Yu

**Affiliations:** Maternal and Child Health Bureau, Health Resources and Services Administration, US Department of Health and Human Services, Room 18-41, 5600 Fishers Lane, Rockville, MD 20857, USA

## Abstract

We examined the prevalence and correlates of parent-reported behavioral problems among immigrants and US-born children aged 6–17 years. The 2007 National Survey of Children's Health was used to develop an 11-item factor-based behavioral problems index (BPI) and a dichotomous serious behavioral problems (SBP) measure. Logistic and least-squares regression and disparity indices were used to analyze differentials. BPI scores varied from 92.3 for immigrant Asian children to ≥102.4 for native Hispanic and Black children. The prevalence of SBP ranged from 2.9% for immigrant Asian children to 17.0% for native Black children. Children in most ethnic-immigrant groups had higher adjusted levels of behavioral problems than immigrant Asian children. Native Hispanic children, native and immigrant White children, immigrant Black children, and native Asian children had ≥3.0 times higher adjusted odds of SBP than immigrant Asian children. Lower socioeconomic status, obesity, physical inactivity, lack of sports participation, increased television viewing, and sleep disruption were associated with greater behavioral problems. Sociodemographic and behavioral factors accounted for 37.0% and 48.5% of ethnic-immigrant disparities in BPI and SBP, respectively. Immigrant children had fewer behavioral problems than native-born children. Policies aimed at modifying obesity-related behaviors and social environment may lead to improved behavioral/emotional health in both immigrant and native children.

## 1. Introduction

Behavioral and emotional problems in children have significant impacts on their health and wellbeing [1–4]. Children with emotional and behavioral problems are more likely to have poor academic performance, to repeat a grade in school, to face school suspension, and to develop behavioral and mental health problems in adulthood and are less likely to engage in social activities outside of school [1–4]. Evidence also suggests that emotional and behavioral problems in children have an adverse effect on their parents' mental health and subjective wellbeing and are associated with increased levels of parental and familial stress [1, 2, 5].

A number of studies have analyzed gender, racial/ethnic, and socioeconomic variations in children's mental health and behavioral problems [3–9]. However, few studies have examined the impact of nativity/immigrant status, obesity, and obesity-related risk factors on behavioral outcomes among children [10–13]. Moreover, most previous studies on ethnic and social determinants have focused on either internalizing (depression, anxiety, and withdrawal) [3] or externalizing behaviors (aggression and antisocial behavior) [7], but few have examined determinants of problem behaviors more broadly capturing both internalizing and externalizing behaviors [4, 8–10]. Furthermore, most studies of emotional/behavioral outcomes have focused either on young children [3, 7, 8] or on adolescents [9, 10] but have generally not covered the entire school-age population of 6-to-17 years old [4]. Additionally, most studies of obesity-related effects on children's mental health have used nonnationally representative samples [4].

The immigrant population in the United States has grown considerably in the last four decades. In 2011, there were 40.4 million immigrants in the United States, which indicates an increase of 30.8 million in the immigrant population since 1970 [14–19]. Immigrants currently account for 13.0% of the total US population [15, 19] increase in the immigrant child population has been even more rapid and substantial. The number of US children in immigrant families increased more than two-fold in the past two decades, from 8.2 million in 1990 to 17.5 million in 2011 [19, 20]. In 2011, 24.4% of US children had at least one foreign-born parent [19, 20]. Despite the rapid increase in the population, behavioral health disparities among immigrant children are not well studied. It is often hypothesized that children in immigrant families may be at greater risk for behavioral and emotional health problems than children of native- or US-born parents due to the stresses associated with immigration, acculturation, and their ethnic-minority status [10, 14].

Obesity-related behaviors are one possible mechanism through which ethnic and social factors might influence behavioral and mental health disparities in children [4, 11]. Assessing potential mental health effects of obesity-related risks is important because the prevalence of childhood obesity and physical inactivity has risen markedly during the past three decades not only in the United States, but also in many other industrialized countries of the world [21, 22]. Moreover, obesity-related behaviors are amenable to change through public policy and social interventions [21–23]. To the extent that obesity-related risks negatively affect children's behavioral health, positive changes in these risk behaviors are expected to improve mental health outcomes in children [4].

To address the aforementioned gaps in behavioral health research, we used the 2007 National Survey of Children's Health (NSCH) to analyze the impact of ethnic-immigrant status and several obesity-related risk factors on childhood behavioral problems in the United States [24, 25]. In this study, we (1) estimate prevalence of behavioral problems among 62,804 children and adolescents aged 6–17 by ethnic-immigrant status, obesity, physical activity, sedentary activities, sleep behavior, and other sociodemographic characteristics, (2) assess the effects of ethnic-immigrant status and obesity-related risk factors on childhood behavioral problems after adjusting for household-level and individual-level socioeconomic and demographic characteristics, and (3) examine the extent to which behavioral health effects of obesity-related risk factors vary by the immigrant status.

## 2. Methods

The data for this study came from the 2007 NSCH [24–26]. The survey was conducted by the Centers for Disease Control and Prevention's (CDC) National Center for Health Statistics (NCHS), with funding and direction from the Health Resources and Services Administration's Maternal and Child Health Bureau [24, 25]. Its purpose was to provide national and state-specific prevalence estimates for a variety of indicators of children health and wellbeing [24–26].

The survey was conducted by telephone between April 2007 and July 2008 [25, 26]. It had a total sample size of 91,642 children under 18 years of age, including a sample of about 1800 children per state [25, 26].A random-digit-dial sample of households with children younger than 18 was selected from each of the 50 states and Washington, DC. One child was selected in each identified household to be the subject of the survey.

Interviews were conducted in English, Spanish, and four Asian languages. The respondent was the parent or guardian who knew most about the child's health status and health care. Consequently, all survey data were based on parental reports. The interview completion rate, measuring the percentage of completed interviews among known households with children, was 66.0% [26]. The overall response rate at the national level was 46.7% [26]. Substantive and methodological details of the survey are described elsewhere [24–26]. The NCHS Research Ethics Review Board approved all data collection procedures.

The dependent variable was measured by a composite behavioral problems index (BPI). Behavioral problems scales have been used previously and have been validated against emotional/behavioral and school outcomes among children [4, 27–29]. In our study, the BPI was constructed using principal components analysis of 11 items capturing parents' ratings of their children on a set of behaviors, including arguing, bullying, disrespect, not getting along with others, disobedience, irritability, lacking empathy and conflict resolution strategies, and feelings of worthlessness, depression, and detachment (Table 1) [4]. The factor loadings for the BPI varied from a low of 0.51 for “detachment” to a high 0.63 for “irritability.” The BPI had a high reliability coefficient (alpha = 0.80). Higher scores on the BPI (mean = 100; SD = 20) indicate higher levels of behavioral problems [4].

The validity of the BPI was assessed by comparing the item factor loadings for immigrants and natives and for major racial/ethnic groups. The factor loadings for the various ethnic and immigrant groups were generally similar in relative importance and the reliability coefficient of the BPI for each subgroup was high, exceeding 0.75. A binary variable of serious behavioral problems (SBP) was also defined based on the 90th percentile cutoff for the BPI scores. Such cutoffs have been used previously to identify children with serious behavioral problems [29]. To test the concurrent validity of the BPI, we estimated the magnitude of the association (the gamma statistic) between the dichotomous SBP variable and parent-reported diagnoses of depression (0.88 for both immigrants and natives), anxiety (0.82 for immigrants and 0.81 for natives), oppositional defiant disorder/conduct disorder (0.94 for immigrants and 0.93 for natives), and Attention Deficit Disorder (ADD) or Attention Deficit Hyperactive Disorder (ADHD) (0.72 for both immigrants and natives). The quartile distribution of the BPI was also highly correlated with the degree/severity of depression (*γ* = 0.83 for immigrants and 0.81 for natives), anxiety (0.73 for immigrants and 0.69 for natives), conduct disorder (0.91 for immigrants and 0.89 for natives), and ADD/ADHD (0.56 for immigrants and 0.54 for natives). To test the predictive validity of the BPI, we examined its association with several child and parental health outcomes. Higher BPI levels were related with higher prevalence and risks of poor child health, school absence, and poor mental health, unhappiness, and higher stress/aggravation levels among parents in both immigrant and native-born families (Table 2).

The primary covariates of interest were nativity/immigrant status, obesity/overweight status, and obesity-related risk factors. Immigrant status was defined according to parents' nativity status [14, 30, 31]. Immigrant children and adolescents were defined as those born to one or both immigrant parents. US-born children with both US-born parents were considered as the native-born [14, 30, 31]. Race/ethnicity was classified into 5 categories: non-Hispanic whites, non-Hispanic blacks, Hispanics, Asians, and other (including American Indians/Alaska Natives, Hawaiian/Pacific Islanders, and mixed/multiple races). The joint variable of ethnic-immigrant status included 10 categories, with each of the 5 broad ethnic groups having two nativity categories. Acculturation was measured by the number of years that the foreign-born mother lived in the United States.

The BMI status consisted of three categories: overweight (BMI = 85th to <95th percentile), obese (BMI =≥ 95th percentile), and normal weight (BMI < 85th percentile). Obesity-related factors included television viewing time, physical activity, sports participation, and sleep duration. Television viewing was based on the question, “on an average school day, how many hours does the child usually watch TV, watch videos, or play video games?” The number of hours spent per day watching television was coded into four categories: <1, 1, 2, and ≥3. Physical activity (PA) was based on the question, “During the past week, on how many days did the child exercise or participate in physical activity for at least 20 minutes that made him/her sweat and breathe hard, such as basketball, soccer, running, or similar aerobic activities?” The responses to the PA variable were coded as 0, 1-2, 3-4, and ≥5 days per week. Sport participation was a dichotomous variable derived from the question, “During the past 12 months, was the child on a sports team or did s/he take sports lessons after school or on weekends?” Sleep behavior was measured by the number of nights the child got adequate sleep during the week preceding the survey and was coded as 0, 1–4, and 5–7 nights per week [4].

Besides the above primary covariates, based on previous research, we used the following variables as covariates: child's age, gender, race/ethnicity, household composition, perceived neighborhood safety, and household poverty status (measured as a ratio of family income to the poverty threshold) [1–5, 11, 13, 27–29, 32, 33]. These covariates were measured as shown in Tables 3–5.

Fewer than 2% of the observations had missing data on one or more of the behavioral items comprising the BPI, which was constructed for 62,804 children aged 6–17. Household income was imputed for 9% of the observations by using a multiple imputation technique [26]. For all other covariates, there were few missing cases, which were excluded from multivariable models, yielding an effective sample size of 60,787 for the fully adjusted covariate models.

 The *χ* ^2^ statistic was used to test the overall association between each covariate and behavioral problems. To estimate differentials in risks of SBP and mean BPI scores, we fitted two sets of logistic and least squares regression models. The first set of models present the unadjusted odds of serious behavioral problems or mean BPI associated with each covariate. The second set of models yield the adjusted effects of immigrant status after controlling for age, gender, race/ethnicity, household composition, neighborhood safety, poverty status, and obesity-related behaviors. A demographic model without the intervening obesity-related behaviors was also fitted, which yielded the underlying adjusted effects of ethnic-immigrant status and SES.

We used root mean square deviation (RMSD) as a new summary measure of behavioral health disparities among 10 ethnic-immigrant groups. The RMSD is similar to the square root of the variance, except that the average squared deviations are calculated using a “standard” estimate other than the sample mean. The RMSD is given by the formula

(1) $$\begin{array}{c} \text{RMSD}=\text{SQRT}\left\{\frac{\sum_{i}^{}{\left(X_{ri}-\;X_{rl}\right)}^{2}}{I}\right\}, \end{array}$$

where *X*_*ri*_ is the BPI score or SBP prevalence for the *i*th group (*i* = 1,2,…, 10), *X*_*rl*_ is the corresponding statistic for the “standard” group (total US population) or group with the lowest prevalence of behavioral problems (i.e., immigrant Asian children), and *I* is the number of ethnic-immigrant groups (10) being compared.

While RMSD is a measure of absolute health disparity, the coefficient of variation (CV) of the RMSD provides an estimate of relative disparity and is given by

(2) $$\begin{array}{c} \text{CV}\left(\text{RMSD}\right)=\left(\frac{\text{RMSD}}{X_{rl}}\right)\times 100;\;X_{rl}>0. \end{array}$$

An additional measure of relative disparity, population attributable risk (PAR) indicating excess behavioral problems was also computed. We estimated interactions of obesity-related risk factors with gender and immigrant status by running separate regression models for immigrant and native children and for male and female children. The results of stratified models are briefly discussed but are not shown to conserve space. To account for the complex sample design of the NSCH, SUDAAN software was used to conduct logistic and least squares analyses and to estimate means, prevalence estimates, predicted marginals, and corresponding standard errors [34].

## 3. Results

The 10 ethnic-immigrant groups varied substantially in their sociodemographic and behavioral characteristics (Table 3). Immigrant children in each racial/ethnic group were more likely to live in two-parent households than native-born children. Approximately 40% of immigrant Hispanic children or parents lived below the poverty line, compared with 6.4% of white immigrants and 5.7% of native-born Asians. Immigrant white and Black children had higher household socioeconomic status (SES) than their native-born counterparts, while the converse was true for Hispanic children. More than half of all immigrant children lived in non-English-speaking households, compared with 2.9% of native-born children. Approximately one-fourth of native-born Black children and immigrant Hispanic children lived in unsafe neighborhoods, compared with 6.8% of immigrant white children. Immigrant children were more likely to be physically inactive than native-born children, with immigrant Hispanic children twice as likely to be inactive as native-born Hispanic children. Television viewing and sleep problems were less common among immigrant children than native children. Immigrant children had lower obesity and overweight prevalence than native children. Obesity prevalence varied from a low of 11.9% for immigrant white children to a high of 32.9% for native-born Black children (Table 3). Immigrant children were significantly less likely to have access to mental health services than native-born children (44.5% versus 36.2%).

In 2007, 11.8% of US children aged 6–17 were estimated to have SBP, with the prevalence being 30% higher among males than females (Table 4). The overall prevalence of SBP in immigrant children was 10.6%, not significantly different from the prevalence of 12.0% in native children. Mean BPI level was 98.1 for immigrant children, significantly lower than the mean score of 101.5 for native children. Nativity differentials in mean BPI were significant for both males and females (*P* = 0.0003); the mean BPI scores were 99.3, 102.7, 96.9, and 100.2 for immigrant males, native males, immigrant females, and native females, respectively. Immigrant patterns in mean BPI score and SBP prevalence varied by ethnicity. The BPI score varied from a low of 92.3 for immigrant Asian children to 102.4 or higher for native Hispanic and Black children. Prevalence of SBP ranged from 2.9% for immigrant Asian children to 17.0% for native Black children (Table 4). After adjusting for sociodemographic and obesity-related risk factors, children in most ethnic-immigrant groups had higher levels of behavioral problems than immigrant Asian children (Table 5). The mean BPI scores for native Hispanic children, native and immigrant white children, and native Asian children were at least 5.3 points higher than those for immigrant Asian children. Native Hispanic children, native and immigrant white children, immigrant Black children, and native Asian children had at least 3.0 times higher adjusted odds of experiencing SBP than immigrant Asian children (Table 5).

Children's risk of SBP and mean levels of BPI increased significantly in relation to mother's duration of residence in the United States (Table 5). Immigrant children whose mothers had immigrated to the US in the past 5 years scored 3.7 points lower on the mean BPI and experienced 54% lower odds of SBP than children of US-born parents (Table 5).

About 23% of children who did not engage in any vigorous PA experienced SBP, compared to 8.8% of children who exercised at least five days/week. Children with no PA had 2.0 times higher adjusted odds of SBP than physically active children, with mean BPI scores increasing in relation to lower PA levels (Table 5). Children who did not participate in sports had 64% higher odds of SBP than children who did participate. Children who watched television >2 hours/day had 65% higher adjusted odds of SBP, with mean levels of behavioral problems increasing in accordance with higher amounts of television viewing. Sleep duration was strongly and inversely associated with behavioral problems in children. Children experiencing inadequate sleep during the entire week had 3.2 times higher odds of SBP than children who did not experience any sleep problems during the week. The adjusted mean BPI scores among children increased consistently in relation to the frequency of sleep problems (Table 5). Obese children had significantly higher BPI levels than normal-weight children (Table 4). However, obesity or overweight status did not have a statistically significant effect on childhood behavioral problems after adjusting for sociodemographic and behavioral covariates.

About 20% of children in unsafe neighborhoods experienced SBP, compared with 10.5% of children in safe neighborhoods. After adjusting for covariates, children in unsafe neighborhoods had 47% higher odds of SBP and 3.7 higher mean BPI than children living in safe neighborhoods. The impact of household income on children's behavioral problems was large. Nearly 21% of poor children experienced SBP, compared with 6.6% of affluent children (Table 4). After adjusting for the covariates, children living below the poverty line had 2.1 times higher odds of SBP and 7.8 points lower mean BPI than children whose family income exceeded 400% of the poverty threshold (Table 5). Children in stepfamily and single-mother households, respectively, had 2.0 and 1.6 times higher odds of SBP than children in two-biological-parent households even after adjusting for sociodemographic and behavioral characteristics (Table 5). All covariates including sociodemographic characteristics and obesity-related risk factors together accounted for 10.5% of the variance in the BPI (Table 6).

Interactions of gender with immigrant status, SES, and obesity-related behaviors were not statistically significant. Ethnic-immigrant and obesity-related behavioral patterns in BPI and SBP were generally similar for males and females. Similarly, socioeconomic and behavioral patterns in SBP and BPI were similar for immigrants and natives, with lower SES, unsafe neighborhoods, single-mother and stepfamily-households, physical inactivity, lack of sports participation, and higher levels of screen time and sleep disruption being associated with greater behavioral problems in both immigrant and native children.

## 4. Discussion

Using data from a large, nationally representative survey in the United States, we found substantial differentials in children's behavioral/emotional problems according to their ethnic-immigrant status. Overall, immigrant children were not more likely to have serious behavioral problems than native children, contrary to the commonly believed notion in the literature [10, 14]. In fact, mean levels of behavioral problems were significantly lower in immigrant children than in native children. Immigrant Asian and Hispanic children had lower BPI levels than their native-born counterparts of equivalent socioeconomic and behavioral background. However, in keeping with the acculturation hypothesis, behavioral health problems among children from immigrant families seem to increase with increasing duration of residence in the United States [30, 31]. Moreover, their behavioral health advantage notwithstanding many immigrant children appear to have a much higher unmet need for mental health services than native children, as reported in Table 3.

Besides the substantial effect of ethnic-immigrant status, we found that children and adolescents with higher levels of physical inactivity, TV viewing, and sleep problems had significantly higher levels of behavioral problems and a higher likelihood of experiencing serious behavioral problems [4]. The effects of obesity-related risk factors on children's behavioral problems remained significant, albeit somewhat reduced, after controlling for household socioeconomic and demographic characteristics. The substantial impact of household SES and perceived neighborhood safety on children's behavioral health is consistent with the findings of past research [4–6, 29].

We examined possible mechanisms through which ethnic-immigrant status and household socioeconomic factors might influence children's risks of experiencing behavioral problems. Increased TV viewing, lack of physical activity and sports participation, and sleep disruption were each independently related to BPI and SBP risk in children. Assessment of the effects of these behavioral risk factors on children's mental health has received little attention in past research [4, 33]. We are not aware of any research that looks at the impact of these characteristics in explaining behavioral health disparities among immigrant and native-born children.

Although household socioeconomic, demographic, and behavioral factors accounted for a substantial proportion of ethnic-immigrant disparities in behavioral health, ethnic-immigrant differentials in behavioral health remained marked. Adjustment for socioeconomic, demographic, and obesity-related factors reduced the magnitude of ethnic-immigrant disparities in SBP by 48.5% and in mean BPI by 37% (Table 6). What might explain the residual differences?

Positive immigrant selectivity in health, education, skills, and ambition and higher levels of social support have been suggested as likely explanations for better health of immigrants [35–37]. More than 80% of immigrants to the United States come from Latin America and Asia [16], who appear to be a healthier group than those who remain in their countries of origin. Given the US immigration laws of the past four decades, most legal immigrants today are chosen rather than randomly self-selected based primarily on their skill criteria [35–37].

Several other factors that we did not consider might account for the observed ethnic-immigrant differentials in behavioral health. They include factors such as parental physical and mental health status, socioeconomic characteristics other than household income, social environmental factors, including neighborhood social and built environments, ethnic and cultural differences in interpretation or understanding of survey questions including those related to perceived childhood behaviors, and interviewer bias [4]. Future research is needed to examine the role of these factors in explaining behavioral health disparities reported here.

A major strength of our study concerns estimating the effects of a number of obesity-related risk factors and detailed ethnic-immigrant status on children's behavioral problems—which have been studied either little or not at all. The construction of a highly valid and reliable behavioral problems index for various racial/ethnic and immigrant groups and of a binary variable capturing serious behavioral problems in US children should be seen as an important contribution to the behavioral health literature. Measuring various types of child and parental health effects of childhood behavioral problems for both immigrants and natives is a unique and novel contribution. The other strengths of our study include the large sample size, the generalizability of our findings, and examination of whether behavioral health effects of obesity-related factors and household SES vary by nativity/immigrant status.

This study has limitations. First, behavioral problems in our study were based on parental reports and might not accurately reflect the true prevalence, particularly among older adolescents or among those primarily experiencing internalizing symptoms [4]. Second, our study lacked data on other immigration-related variables such as citizenship, naturalization, and legal status that may affect the health and socioeconomic status of both parents and children in immigrant families [30, 31, 35–37]. Third, the NSCH lacks ethnic detail. The survey does not identify specific immigrant Hispanic and Asian subgroups such as Mexicans, Cubans, Puerto Ricans, Central and South Americans, Chinese, Asian Indians, Filipinos, Japanese, Koreans, and Vietnamese. These groups are quite heterogeneous in their socioeconomic, cultural, and immigration characteristics and are, therefore, expected to differ in their health and behavioral outcomes as well [35]. Fourth, immigrant parents in socioeconomically disadvantaged families may have downgraded the severity or frequency of behavioral problems among their children, thus creating an “optimistic” bias [4]. Fifth, because of the cross-sectional nature of the NSCH, causal inferences about the relationship between social and obesity-related risk factors and childhood behavioral problems cannot be drawn [4].

The evidence presented here suggests that ethnic-immigrant status, obesity-related risk factors, and adverse socioeconomic conditions are significantly associated with increased risk of behavioral problems in children. Since obesity-related behaviors are amenable to change through social and public policy interventions, these behaviors along with household socioeconomic conditions remain crucial in determining children's emotional and behavioral health. Since the social environments, including household SES and neighborhood conditions, are the underlying determinants of obesity-related risk factors [21–23], social policy measures aimed at improving the broader social environments are vital to improving the overall health of immigrant and native children in general and their mental health in particular. Achieving health equity continues to be one of the most important overarching goals for the United States [38]; however, the existence of substantial socioeconomic inequalities among ethnic-immigrant groups, as shown in this study, poses a tremendous challenge to achieving improvements in the mental health of children in several immigrant and native-born groups.

## Figures and Tables

**Table 1:** Factor loadings and factor score coefficients (derived from the principal components analysis) for 11 variables comprising the behavioral problems index, US children aged 6–17 years: the 2007 National Survey of Children's Health.

Variable	All races Total population		Non-Hisp. White	Non-Hisp. Black	Hispanic	Asian	Mixed or multiple race	Immigrants	Native born
	Factor loadings	Factor score coefficients	Factor loadings	Factor loadings	Factor loadings	Factor loadings	Factor loadings	Factor loadings	Factor loadings
Child argues too much	0.61	0.16	0.60	0.61	0.60	0.52	0.63	0.58	0.61
Child bullies or is cruel or mean to others	0.61	0.16	0.60	0.66	0.62	0.52	0.62	0.58	0.62
Child does not show respect for teachers and neighbors	0.52	0.14	0.52	0.57	0.45	0.45	0.56	0.42	0.53
Child does not get along with other children	0.60	0.16	0.60	0.63	0.54	0.50	0.60	0.52	0.60
Child is disobedient	0.61	0.16	0.61	0.61	0.62	0.63	0.62	0.60	0.61
Child is stubborn, sullen, or irritable	0.63	0.17	0.63	0.64	0.65	0.64	0.64	0.64	0.63
Child does not try to understand other people's feelings	0.59	0.16	0.61	0.57	0.50	0.59	0.61	0.53	0.60
Child does not try to resolve conflicts with classmates/family/friends	0.52	0.14	0.56	0.48	0.41	0.45	0.54	0.46	0.53
Child feels worthless or inferior	0.57	0.15	0.59	0.54	0.54	0.54	0.61	0.54	0.58
Child is unhappy, sad, or depressed	0.61	0.16	0.61	0.61	0.63	0.57	0.64	0.60	0.61
Child is withdrawn and does not get involved with others	0.51	0.14	0.53	0.50	0.49	0.43	0.55	0.49	0.52

Proportion of total variance explained by factor	0.34		0.35	0.34	0.31	0.29	0.37	0.30	0.34
Cronbach's alpha (reliability coefficient)	0.80		0.81	0.81	0.77	0.75	0.83	0.76	0.81
Sample size* (N) *	62,804		43,328	6,281	7,025	1,398	2,729	7,695	55,109

Each variable is a 5-category item with response codes 1 (never), 2 (rarely), 3 (sometimes), 4 (usually), and 5 (always).

**Table 2:** Predictive validity of the behavioral problems index (BPI) showing its impact on child and parental health among immigrants and the native-born: the 2007 National Survey of Children's Health.

	Prevalence (%)^2^	SE	Odds ratio	95% CI	Prevalence (%)^2^	SE	Odds ratio	95% CI	Prevalence (%)^2^	SE	Odds ratio	95% CI
	Fair or poor child health status				School absence > 2 weeks				Child's lack of social participation			

Immigrants (behavioral problems index												
(mean index score = 100; SD = 20^1^)												
66.27–85.87	3.63	1.10	1.00	Reference	2.17	0.79	1.00	Reference	48.56	2.74	1.00	Reference
85.88–98.00	6.51	1.84	1.85	0.78–4.35	2.61	0.94	1.21	0.43–3.39	48.59	3.04	1.00	0.73–1.38
98.01–125.34	6.97	1.43	1.99	0.93–4.23	2.51	0.41	1.16	0.52–2.60	52.86	2.53	1.19	0.89–1.59
125.35–232.27	18.85	4.04	6.16	2.75–13.78	8.09	2.73	3.97	1.42–11.12	63.53	4.15	1.84	1.22–2.79

Native born (behavioral problems index)												
66.27–85.87	1.95	0.40	1.00	Reference	4.30	0.54	1.00	Reference	38.03	1.07	1.00	Reference
85.88–98.00	1.87	0.26	0.96	0.58–1.57	5.62	0.53	1.32	0.96–1.83	35.25	0.95	0.89	0.79–1.00
98.01–125.34	2.70	0.32	1.40	0.87–2.25	6.39	0.45	1.52	1.13–2.04	39.70	0.84	1.07	0.96–1.20
125.35–232.27	8.29	0.92	4.55	2.83–7.31	12.65	0.99	3.22	2.36–4.40	57.75	1.53	2.23	1.91–2.59

	Fair or poor maternal health status				Fair or poor paternal health status				Parents unhappy with each other			

Immigrants (behavioral problems index)												
66.27–85.87	1.54	0.37	1.00	Reference	1.52	0.38	1.00	Reference	13.22	1.61	1.00	Reference
85.88–98.00	6.14	1.36	4.18	2.15–8.13	5.40	1.47	3.70	1.75–7.82	17.88	2.22	1.43	0.95–2.14
98.01–125.34	9.02	1.64	6.33	3.42–11.73	9.34	1.69	6.67	3.56–12.50	29.36	2.63	2.73	1.88–3.95
125.35–232.27	26.34	4.24	22.86	12.04–43.40	18.33	3.71	14.53	7.28–29.01	33.30	4.37	3.28	2.04–5.26

Native born (behavioral problems index)												
66.27–85.87	3.43	0.36	1.00	Reference	2.80	0.59	1.00	Reference	10.14	0.73	1.00	Reference
85.88–98.00	4.02	0.36	1.18	0.89–1.56	3.19	0.57	1.14	0.66–1.99	14.28	0.73	1.48	1.21–1.80
98.01–125.34	7.52	0.44	2.29	1.79–2.92	5.08	0.49	1.86	1.17–2.96	19.14	0.70	2.10	1.75–2.51
125.35–232.27	23.57	1.56	8.68	6.62–11.38	14.06	1.67	5.68	3.44–9.37	36.28	1.90	5.04	4.03–6.32

	Parental stress or aggravation^3^				Parents not coping well with daily demands of parenting							

Immigrants (behavioral problems index)												
66.27–85.87	7.23	1.76	1.00	Reference	28.19	2.40	1.00	Reference				
85.88–98.00	6.87	1.60	0.95	0.46–1.92	35.32	2.70	1.39	1.00–1.93				
98.01–125.34	12.69	1.75	1.86	1.02–3.40	49.21	2.56	2.47	1.82–3.36				
125.35–232.27	38.72	4.57	8.11	4.29–15.33	58.88	4.56	3.65	2.36–5.64				

Native born (behavioral problems index)												
66.27–85.87	2.21	0.23	1.00	Reference	23.59	0.83	1.00	Reference				
85.88–98.00	3.70	0.41	1.70	1.25–2.32	37.77	0.98	1.97	1.74–2.22				
98.01–125.34	8.71	0.51	4.23	3.31–5.41	51.63	0.84	3.46	3.09–3.87				
125.35–232.27	41.15	1.60	30.99	24.19–39.69	69.01	1.56	7.21	6.09–8.55				

^1^Higher scores on the index indicate higher levels of behavioral problems. The first BPI category is the first quartile with fewest behavioral problems, the second represents the second quartile, the third catergory is median to <90 percentile, and the fourth category is ≥90th percentile reflecting serious behavioral problems.

^2^Differences in the prevalence of each health outcome across the BPI categories were statistically significant at *P* < 0.05. ^3^A composite variable denoting parenting aggravation if parents reported in the past month that child was much harder to care for, or were bothered by child's behavior, or felt angry with their child.

**Table 3:** Selected sociodemographic and behavioral characteristics (%) of US children aged 6–17 years in 10 ethnic-immigrant groups: the 2007 National Survey of Children's Health (*N* = 62,804).

Characteristic	Hispanic children of		NH white children of		NH black children of		Asian children of		Other children	
	Immigrant parents	US-born parents	Immigrant parents	US-born parents	Immigrant parents	US-born parents	Immigrant parents	US-born parents	Immigrant parents	US-born parents^1^
Number in sample	3,189	3,836	2,518	40,810	460	5,821	894	504	634	4,138
Percentage of total US population	9.80	8.78	3.74	53.24	1.51	13.33	2.50	0.64	0.89	5.57
Two-parent households^2^	81.71	40.46	85.60	69.36	66.79	28.65	91.94	56.96	85.59	44.42
Single-mother households	5.44	38.74	2.51	14.39	10.20	46.97	2.65	31.20	1.97	27.91
Non-English speaking households	71.01	20.99	5.86	0.26	11.18	0.51	40.86	7.30	5.84	4.42
Unsafe neighborhood	24.60	19.39	6.78	6.91	19.29	26.01	15.27	8.97	12.13	15.28
Households below poverty level	39.58	27.26	6.41	8.38	12.96	32.49	9.29	5.73	6.71	18.45
Households with income at or above 400% of poverty level	8.60	20.01	48.59	38.02	28.42	14.02	51.00	46.80	47.59	27.34
Parental education ≥16 years	17.68	24.02	66.99	49.11	57.86	22.65	74.04	79.85	61.22	35.51
TV watching >2 hours/day	25.11	24.40	12.32	17.26	26.45	39.74	7.43	18.75	22.42	24.56
No physical activity	24.12	12.64	7.00	7.01	7.13	13.38	6.13	5.48	6.85	9.40
Lack of sports participation	60.32	52.55	31.34	34.57	34.49	51.44	36.08	30.15	37.34	40.95
Inadequate sleep <5 nights/week	9.86	12.49	10.44	14.07	11.84	15.78	13.30	11.46	11.76	12.78
Obesity prevalence^3^	28.11	27.34	11.88	16.61	17.52	32.90	12.35	12.74	10.04	23.80
Overweight prevalence^4^	45.21	42.19	27.50	31.06	35.56	48.21	23.68	28.71	31.04	41.69
Did not receive needed mental health treatment	49.68	40.74	42.40	33.75	22.45	44.54	54.44	52.90	23.24	27.23

All chi-square tests for the association between ethnic-immigrant status and each of the demographic characteristics were statistically significant at *P* < 0.01.

^1^Consists primarily of Hawaiian/Pacific Islander children, American Indian/Alaska Native children, mixed race children, and other children.

^2^Includes biological and adoptive parents. ^3^BMI ≥ 95th percentile. ^4^BMI ≥ 85th percentile. NH: non-Hispanic.

**Table 4:** Mean behavioral problems index values and prevalence and unadjusted odds of serious behavioral problems according to ethnic-immigrant status and sociodemographic and behavioral characteristics, US children aged 6–17 years: the 2007 National Survey of Children's Health (*N* = 62,804).

Covariate	Behavioral problems index^1^			Serious behavioral problem^2^			
	Mean	SE	*P* value	Weighted prevalence (%)^3^	SE	Unadjusted OR	95-percent confidence interval
Total population	100.0	0.22		11.76	0.34		
Age (years)							
6–9	100.4	0.36	Reference	9.79	0.53	1.00	Reference
10-11	100.5	0.57	0.865	11.53	0.88	1.20	0.98−1.48
12–14	101.4	0.43	0.074	12.50	0.63	1.32	1.12−1.55
15–17	101.1	0.47	0.267	13.67	0.74	1.46	1.23−1.73
Gender							
Male	102.0	0.31	<0.001	13.26	0.50	1.35	1.19−1.53
Female	99.6	0.32	Reference	10.19	0.45	1.00	Reference
Ethnic-immigrant status							
Hispanic children of immigrant parents	99.4	0.99	<0.001	13.21	1.55	5.05	2.48−10.31
Hispanic children of US-born parents	102.4	1.03	<0.001	14.26	1.55	5.52	2.72−11.19
White children of immigrant parents	99.2	0.89	<0.001	8.74	1.16	3.18	1.54−6.53
White children of US-born parents	100.9	0.24	<0.001	10.13	0.37	3.74	1.92−7.29
Black children of immigrant parents	98.4	2.18	0.012	12.17	3.27	4.60	1.88−11.23
Black children of US-born parents	102.6	0.68	<0.001	16.95	1.00	6.77	3.44−13.32
Asian children of immigrant parents	92.3	1.10	Reference	2.93	0.96	1.00	Reference
Asian children of US-born parents	99.8	2.41	0.004	9.63	3.60	3.53	1.24−10.07
Other children of immigrant parents	96.2	2.13	0.104	8.33	1.96	3.02	1.31−6.93
Other children of US-born parents	103.4	0.82	<0.001	15.08	1.56	5.89	2.91−11.91
Mother's duration of residence in the US (years)							
<5	98.7	2.05	0.225	9.41	2.88	0.81	0.41−1.57
5–9	99.0	1.48	0.147	13.19	3.10	1.18	0.69−2.02
10–14	97.2	1.53	0.009	10.95	2.31	0.95	0.60−1.53
15+	99.1	0.88	0.021	11.19	1.28	0.98	0.75−1.27
US born	101.2	0.24	Reference	11.41	0.36	1.00	Reference
Child's race/ethnicity							
Non-Hispanic White	100.8	0.23	<0.001	10.04	0.36	2.49	1.48−4.19
Non-Hispanic Black	102.2	0.65	<0.001	16.46	0.96	4.40	2.58−7.50
Hispanic	100.8	0.71	<0.001	13.71	1.10	3.54	2.05−6.12
American Indian/Alaska native	102.7	1.45	<0.001	13.76	2.36	3.56	1.86−6.79
Asian	93.8	1.05	Reference	4.29	1.08	1.00	Reference
Hawaiian/Pacific Islander	99.4	3.02	0.080	14.56	4.35	3.80	1.61−8.97
Non-Hispanic mixed race	102.7	1.06	<0.001	13.59	1.54	3.51	1.97−6.24
Other	102.2	1.81	<0.001	15.66	4.03	4.14	1.88−9.13
Household composition							
Two-parent biological	98.4	0.26	Reference	8.04	0.36	1.00	Reference
Two-parent stepfamily	105.3	0.74	<0.001	17.78	1.27	2.47	2.03−3.01
Single mother	105.3	0.54	<0.001	18.45	0.94	2.59	2.22−3.02
Other family type	103.3	0.79	<0.001	16.78	1.22	2.31	1.90−2.81
Household poverty status (ratio of family income to poverty threshold)							
<100%	105.7	0.68	<0.001	20.73	1.10	3.73	3.10−4.49
100–199%	102.2	0.59	<0.001	14.87	0.92	2.49	2.06−3.02
200–399%	100.3	0.38	<0.001	10.03	0.56	1.59	1.34−1.89
≥400%	97.9	0.29	Reference	6.55	0.40	1.00	Reference
Perceived neighborhood safety							
Unsafe	106.1	0.72	<0.001	19.89	1.24	2.12	1.79−2.51
Safe	100.0	0.23	Reference	10.48	0.34	1.00	Reference
Television watching (number of hours per day)							
<1	97.7	0.42	Reference	8.10	0.67	1.00	Reference
1	99.4	0.42	0.004	9.48	0.59	1.19	0.95−1.48
2	101.3	0.38	<0.001	11.79	0.62	1.52	1.23−1.87
>2	105.1	0.55	<0.001	18.05	0.85	2.50	2.03−3.08
Physical activity (number of days per week)							
0	107.8	0.94	<0.001	22.90	1.43	3.06	2.54−3.68
1-2	103.9	0.72	<0.001	16.27	1.22	2.00	1.64−2.44
3-4	100.7	0.39	<0.001	10.91	0.65	1.26	1.07−1.48
5 or more	98.8	0.28	Reference	8.84	0.39	1.00	Reference
Sports participation							
Yes	98.8	0.24	Reference	8.18	0.35	1.00	Reference
No	103.8	0.41	<0.001	16.88	0.64	2.28	2.01−2.59
Sleep behavior (number of nights child getting adequate sleep during past week)							
0	112.0	1.56	<0.001	26.69	2.34	3.52	2.74−4.53
1–4	109.8	0.83	<0.001	22.23	1.33	2.77	2.32−3.30
5-6	102.8	0.39	<0.001	11.51	0.65	1.26	1.08−1.47
7	98.1	0.26	Reference	9.36	0.40	1.00	Reference
BMI status of child							
Normal weight (<85th percentile)	99.9	0.28	Reference	10.37	0.41	1.00	Reference
Overweight (85th to <95th percentile)	101.3	0.61	0.041	11.79	0.75	1.15	0.98−1.36
Obese (≥95th percentile)	103.2	0.52	<0.001	14.94	0.86	1.52	1.30−1.78

^1^Higher scores on the index indicate higher levels of behavioral problems. ^2^ This binary outcome variable was defined on the basis of whether or not the child had a BPI score >90th percentile.

^3^The *χ* ^2^ test for the overall association between each covariate and the prevalence of serious behavioral problems was statistically significant at *P* < 0.01.

**Table 5:** Multivariate weighted least squares and logistic regressions showing the impact of ethnic-immigrant status and sociodemographic and obesity-related risk factors on behavioral problems among US children aged 6–17 years: the 2007 National Survey of Children's Health (*N* = 60,787).

Covariate	Behavioral problems index			Serious behavioral problem	
	Adjusted mean^1^	SE	*P* value	Adjusted odds ratio^2^	95% CI
Age (years)					
6–9	101.6	0.36	Reference	1.00	Reference
10-11	101.5	0.55	0.937	1.28	1.02−1.59
12–14	101.0	0.42	0.309	1.25	1.05−1.50
15–17	98.9	0.46	<0.001	1.14	0.93−1.39
Gender					
Male	102.1	0.31	<0.001	1.50	1.31−1.72
Female	99.2	0.31	Reference	1.00	Reference
Ethnic-immigrant status					
Hispanic children of immigrant parents	97.3	1.03	0.149	2.57	1.22−5.40
Hispanic children of US-born parents	100.4	1.08	<0.001	2.99	1.43−6.29
White children of immigrant parents	101.7	0.94	<0.001	3.44	1.62−7.31
White children of US-born parents	102.0	0.27	<0.001	3.14	1.57−6.25
Black children of immigrant parents	98.6	2.12	0.141	3.55	1.37−9.23
Black children of US-born parents	98.6	0.69	0.010	2.83	1.40−5.74
Asian children of immigrant parents	95.1	1.17	Reference	1.00	Reference
Asian children of US-born parents	101.7	2.40	0.013	3.12	1.04−9.36
Other children of immigrant parents	97.8	1.97	0.236	2.87	1.22−6.75
Other children of US-born parents	102.8	0.81	<0.001	3.67	1.78−7.59
Mother's duration of residence in the US (years)					
<5	97.8	2.04	0.070	0.46	0.23−0.95
5–9	97.7	1.50	0.014	0.85	0.49−1.46
10–14	96.4	1.81	0.006	0.76	0.41−1.42
15+	99.0	0.88	0.009	0.80	0.58−1.10
US born	101.5	0.30	Reference	1.00	Reference
Child's race/ethnicity					
Non-Hispanic white	101.6	0.29	0.017	1.78	0.99−3.21
Non-Hispanic black	98.3	0.64	0.909	1.64	0.90−3.01
Hispanic	100.0	0.80	0.263	1.78	1.00−3.18
American Indian/Alaska native	100.6	1.54	0.286	1.66	0.81−3.42
Asian	98.5	1.22	Reference	1.00	Reference
Hawaiian/Pacific Islander	100.8	2.25	0.369	2.80	1.18−6.66
Non-Hispanic mixed race	102.2	0.97	0.019	2.15	1.14−4.05
Other	102.3	2.24	0.139	1.81	0.54−6.04
Household composition					
Two-parent biological	99.2	0.31	Reference	1.00	Reference
Two-parent stepfamily	104.6	0.72	<0.001	1.99	1.60−2.46
Single mother	102.9	0.56	<0.001	1.61	1.33−1.95
Other family type	102.3	0.83	<0.001	1.79	1.43−2.24
Household poverty status (ratio of family income to poverty threshold)					
<100%	103.6	0.67	<0.001	2.07	1.64−2.62
100–199%	101.1	0.58	0.01	1.63	1.31−2.03
200–399%	100.3	0.38	0.044	1.27	1.05−1.52
≥400%	99.3	0.35	Reference	1.00	Reference
Perceived neighborhood safety					
Unsafe	103.9	0.71	<0.001	1.47	1.20−1.79
Safe	100.2	0.24	Reference	1.00	Reference
Television watching (number of hours per day)					
<1	98.7	0.42	Reference	1.00	Reference
1	99.9	0.42	0.048	1.11	0.88−1.39
2	101.1	0.37	<0.001	1.30	1.05−1.62
>2	103.3	0.52	<0.001	1.65	1.32−2.05
Physical activity (number of days per week)					
0	106.0	0.92	<0.001	2.01	1.63−2.49
1-2	103.0	0.66	<0.001	1.56	1.27−1.91
3-4	101.1	0.39	<0.001	1.23	1.04−1.47
5 or more	99.0	0.31	Reference	1.00	Reference
Sports participation					
Yes	99.6	0.26	Reference	1.00	Reference
No	102.3	0.41	<0.001	1.64	1.41−1.90
Sleep behavior (number of nights child getting adequate sleep during past week)					
0	110.8	1.54	<0.001	3.23	2.47−4.22
1–4	109.3	0.84	<0.001	2.77	2.28−3.37
5-6	103.1	0.39	<0.001	1.39	1.18−1.64
7	98.0	0.26	Reference	1.00	Reference
BMI status of child					
Normal weight	100.5	0.30	Reference	1.00	Reference
Overweight	101.2	0.58	0.293	1.04	0.87−1.24
Obese	101.4	0.50	0.139	1.14	0.95−1.36

^1^Adjusted by multivariate least squares regression for child's age, gender, ethnic-immigrant status (or race/ethnicity and mother's duration of residence in the US), household composition, household poverty status, perceived neighborhood safety, TV watching, physical activity, sports participation, sleep behavior, and overweight/obesity status.

^2^Adjusted by multivariate logistic regression for the same covariates as in the least squares model.

**Table 6:** Disparity indices for mean behavioral problems index (BPI) and prevalence (%) of serious behavioral problems (SBP) among us children and adolescents aged 6–17 years by ethnic-immigrant status: the 2007 National Survey of Children's Health.

	Serious behavioral		Serious behavioral		Serious behavioral		Mean behavioral		Mean behavioral		Mean behavioral	
	problem		problem		problem		problem index		problem index		problem index	
Ethnic-immigrant status			Demographic model		Full model				Demographic model		Full model	
	Observed	SE	Adjusted	SE	Adjusted	SE	Observed	SE	Adjusted	SE	Adjusted	SE
	prevalence		prevalence		prevalence		Score		score		score	
Total population	11.76	0.34	11.76	0.34	11.76	0.34	100.00	0.22	100.00	0.22	100.00	0.22

Hispanic children of immigrant parents	13.21	1.55	11.67	1.39	10.21	1.26	99.36	0.99	98.17	1.01	97.34	1.03
Hispanic children of US-born parents	14.26	1.55	11.71	1.35	11.56	1.33	102.37	1.03	100.53	1.08	100.43	1.08
White children of immigrant parents	8.74	1.16	11.83	1.60	12.90	1.70	99.15	0.89	101.25	0.91	101.72	0.94
White children of US-born parents	10.13	0.37	11.75	0.45	11.99	0.47	100.86	0.24	101.95	0.27	101.99	0.27
Black children of immigrant parents	12.17	3.27	11.92	3.19	13.23	3.54	98.38	2.18	98.10	2.06	98.64	2.12
Black children of US-born parents	16.95	1.00	11.93	0.82	11.06	0.77	102.60	0.68	99.41	0.71	98.63	0.69
Asian children of immigrant parents	2.93	0.96	4.21	1.37	4.49	1.43	92.25	1.10	94.43	1.11	95.10	1.17
Asian children of US-born parents	9.63	3.60	12.03	4.18	11.93	4.19	99.82	2.41	101.11	2.41	101.71	2.40
Other children of immigrant parents	8.33	1.96	10.71	2.27	11.18	2.36	96.15	2.13	97.96	2.01	97.80	1.97
Other children of US-born parents	15.08	1.56	13.55	1.40	13.58	1.33	103.41	0.82	102.91	0.84	102.82	0.81

Percent variance explained (*R*-Squared)							0.78		4.42		10.46	
PAR (%)—excess behavioral problem	75.09		64.20		61.82		7.75		5.57		4.90	
Index of absolute disparity—RMSD	9.07		7.32		7.16		7.85		5.67		5.10	
Index of relative disparity—CV (RMSD)	309.67		173.98		159.36		8.51		6.00		5.36	
			Observed versus		Observed versus				Observed versus		Observed versus	
			demographic model		full model				demographic model		full model	
Reduction in absolute disparity (RMSD)%			−19.29		−21.06				−27.77		−35.03	
Reduction in relative disparity (CV-RMSD)%			−43.82		−48.54				−29.49		−37.02	

PAR: population attributable risk. RMSD: root mean square deviation. CV: coefficient of variation.

In calculation of RMSD, the lowest SBP prevalence or mean BPI score (i.e., for immigrant Asian children) is used as the standard or comparison group.
